# Paternal investment, stepfather presence and early child development and growth among Serbian Roma

**DOI:** 10.1017/ehs.2022.14

**Published:** 2022-04-18

**Authors:** Jelena Čvorović

**Affiliations:** Institute of Ethnography, Serbian Academy of Sciences and Arts, Kneza Mihaila 36, Belgrade, Serbia

**Keywords:** Paternal investment, stepfather presence, early child development, height, Roma

## Abstract

Research on paternal investment and child growth and development is limited outside of high-income countries. Using nationally representative data from low-resource Serbian Roma communities, this study examined father investment (direct care), its predictors and the associations between paternal investment, stepfather presence and child physical growth and early development. The sample included 1222 children aged 35–59 months, out of which 235 were living with biological fathers. Child outcomes included height-for-age *Z-*scores, stunting and early child developmental score. Roma paternal investment was relatively low. There was a positive association of father investment and children's height, and no association with developmental score. The presence of father vs. stepfather did not exert any influence on children. Instead, maternal and child characteristics explained both the overall development and height for Roma children. Thus, older children, born to literate, lower parity mothers of higher status and greater investment had better developmental and growth outcomes; girls were the preferred sex, owing to expected fitness benefits. Reverse causality emerged as the most likely pathway through which the cross-sectional association of father direct care with child growth may manifest, such that Roma fathers tend to bias their investment towards taller, more endowed children, because of greater fitness pay-off.

## Graphical abstract

**Figure:**
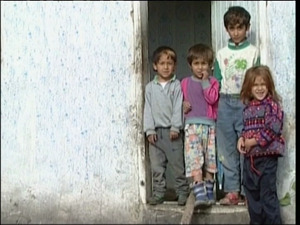

**Social media summary:** Fathers tend to bias their investment towards taller, more endowed children, because of greater fitness pay-off

## Introduction

Parenting investment and practices may be an important determinant of early childhood development, including impact on health outcomes such as growth, nutrition and cognitive and socioemotional development (Francesconi & Heckman, 2016; Frongillo et al., 2017). Parental investment reflects any parental expenditure in terms of time, energy and resources that benefits offspring and thus contributes to their reproductive success (Clutton-Brock, 1991). Among humans, biparental care, where both females and males may invest in children, varies both across and within populations (Prall & Scelza, 2020). It remains uncertain how essential paternal investment was in human evolutionary history (Shenk, 2011; Lawson et al., 2017). Data from different ecological settings suggest that fathers have little influence on their children's welfare, and that investment from fathers may often be substituted with care from others (Sear & Mace, 2008; Sear, 2021). Still, compared with most mammals, human fathers work along with mothers to provide substantial investment for children, thus fatherly care possibly evolved along a human life history strategy of raising energetically costly, slow-developing offspring (Gettler et al., 2020). In addition to the possibility of additional care and protection from the father, the identification of fathers (through marriage) also allows the possibility that humans acquire far more identifiable kin than any other mammal, which may have been important in human cultural and biological evolution (Palmer et al., 2005). Human paternal investment is based on expected fitness returns and dependent on a different range of ecological, cultural and individual charcteristics (Marlowe, 2007). The fitness returns should be responsive to both parental ability to invest and offspring condition, thus under high risk/poor conditions there may be limited differential investment between children, as parents may have little control of their children's survival and reproductive chances (Sear, 2011), or available resources may be directed towards children with more endowments (Hagen et al., 2001; Clutton-Brock, 1991).

Most studies on parental care have focused on mother–child interactions, largely neglecting how the father–child interaction may influence child outcomes (Cano et al., 2019). Current knowledge of the relationship between father investment and care, and child outcomes comes from limited studies conducted mostly in developed countries, focusing on how the quality of father's caregiving impacts children's socioemotional or cognitive development (Frongilio et al., 2017; Sun et al., 2016; Lamb, 2010). In small-scale, traditional societies, studies mostly focused on fathers’ indirect care, such as provisioning and resource defence (Kaplan et al., 2000; Sear & Mace, 2008). Fathers also provide direct care in varying degrees to their offspring, such as early care behaviours and guiding socialisation, depending on local ecology and availability of help from other individuals (Gettler, 2010; Hewlett, 1993; Marlowe, 2007). Recent studies on paternal direct care suggested that variation in paternal care can affect energetic status of children (i.e. weight; Winking & Koster 2015; Boyette et al., 2018). Fathers’ direct care has also been inconsistently associated with child's height, while the means through which direct paternal care may affect a child's growth remain unclear (Maselko et al., 2019; Abate & Belachew, 2017; Dearden et al., 2013; Jeong et al., 2016; Starkweather et al., 2021). A variety of different caregiving pathways have the potential to influence growth, such as that a heritable trait for fathers to invest more could lead to larger size in children, or through a care given in addition to that of mothers and/or by substituting for mothers’ care (Winking & Koster 2015; Starkweather et al., 2021). Other ways through which fathers might influence child growth possibly include giving responsive care and a safe home setting, and decision-making regarding the child's diet (Jeong et al., 2016).

In addition, there is a negative relationship between father absence and indicators of child wellbeing (but see Sear & Mace, 2008). This relationship is even stronger if children live in stepfather households (Emmott & Mace, 2014; Lawson & Mace, 2010). Stepfather presence has been connected with unfavourable effects on multiple child outcomes, such as negative effects on educational attainment, behavioural outcomes and even physical growth, as a result of the reduced quantity and quality of investments children may receive, as stepfathers tend to invest less than fathers, probably because of the insufficient parenting effort within stepfather–child relationship (Case & Paxson, 2001; Lancaster & Kaplan, 2000). Few studies have considered the association between father absence/stepfather presence and child anthropometry as a proxy for health (but see Lawson et al., 2017; Deardenet et al., 2013; Sear et al., 2000).

In ethnic minority, poor populations, children tend to grow up in unfavourable conditions and at risk of poor growth and developmental outcomes, with the risk being even higher for children living in marginalised populations (UNICEF, 2015; Grantham-McGregor et al., 2007; Knauer et al., 2018). Poor parental investment but also father absence can have great costs for the development and growth of children growing up in poverty, as they are exposed to multiple risk factors. Little is known about father investment and how variations in that investment and stepfather presence may contribute to differences in physical growth and development among children from marginalised and stressed populations. This study aims to fill this knowledge gap by assessing paternal investment (direct care) and its predictors, and the link between father investment, stepfather presence and early child development and physical growth in a poor population of Serbian Roma.

The Roma are a diverse population of South Asian origin who migrated to Europe from northwest India between the ninth and the fourteenth centuries. During their exodus and history, the Roma have been persecuted, enslaved, molested, murdered and discriminated against (Čvorović, 2014). Still today, the Roma remain marginalised, exposed to discrimination, poverty and social exclusion. When compared with non-Roma, the Roma experience poorer health across many outcomes, usually attributed to socio-economic differences and access to health care (Cook et al., 2013).

Officially, there are around 140,000 Roma in Serbia, but the estimates run a lot higher (up to 500,000), given the Roma tendency to hide their ethnic origin. Roma, for the most part, have remained a traditional, hard-to-reach population with high levels of unemployment, sub-standard housing, low literacy and skills, and a deepening dependence on state benefits and services (Čvorović, 2014). Roma cultural traditions have encouraged divisions into groups based on descent alone, early endogamous marriages and high fertility. In the past, the traditional Roma occupations, crafts like trough-making, basket-making, spoon-making, blacksmithing, ironsmithing and entertaining (music), were usually passed down from father to son within each group. Traditional oral storytelling served as education for many generations of Roma, as a parental mode of influencing the behaviour of children and, possibly, even distant descendants. In time, Serbian Roma lost the distinctive occupations and have, for the most part, adopted the language and religion of the majority. At present, illiteracy rates remain high: 15% of Roma older than 10 years are illiterate; 21.2% of female Roma are illiterate, with only 33.3% having finished elementary school (Čvorović, 2020a). Within traditional Roma families, women typically have low power in decision-making and control of earnings, while a woman's age and fertility often determine her position. Nevertheless, the gap between the traditional norms and expectations and the real-life behaviours is noticeable: a recent study found that over 60% of males could hardly make ends meet and many could do so only through their wives’ receipt of welfare (Čvorović & James, 2018). Among the Roma, mothers are the primary care-takers of young children, exhibiting lower maternal investment in comparison with non-Roma. Roma infant and child mortality is estimated to be more than double the national average, but owing to the Roma ethnic mimicry, important trends in mortality and morbidity remain hidden. The health status of Roma children is not fully understood yet. The limited studies published on Roma children suggest that children are at risk of underachieving because of poor nutrition and parental care, negatively influencing life outcomes and leading to an intergenerational cycle of poverty and poor development (UNICEF, 2015). In addition, unregistered short-lived unions, divorce and separation are common among Roma, resulting in many Roma children living in a family with one biological parent, usually a mother, who will usually remarry multiple times. Thus for many children, living with the mother's current partner may be a common experience (Čvorović, 2014).

Few studies have considered the relationship between Serbian Roma anthropometrics, health and child outcomes. Adult Roma are well fed, in spite of them belonging to the lowest socio-economic sector of Serbian society, probably as a result of guaranteed income (intergenerational welfare dependency), unemployment and general physical inactivity. Regarding Roma women's stature, height (but not body mass index) reflects a statistically significant heterogeneity despite equal socioeconomic status and similar genetic makeup (Gallagher et al., 2009). Thus, Roma mothers of short stature were found to experience higher rates of child mortality and children in poor health than tall mothers (Čvorović, 2018). Roma children stunting has been found to be unrelated to socioeconomic status, but instead associated with maternal (low) care and (un)registration at birth, while maternal investment towards more endowed children contributes to Roma children's health disparities (Stamenković et al., 2016; Janevic et al., 2010; Čvorović, 2020b).

Nothing is known about the relative importance of Roma paternal investment, its predictors, stepfather presence and the associations with child nutritional and development status. Data from the Multiple Indicator Cluster Surveys rounds 4 and 5 (MICS 4 and 5) for Serbian Roma settlements were combined to assess these issues. Child outcomes included height-for-age *Z-*scores, as anthropometric measures of chronic malnutrition, and the early child developmental score, a combined index of basic learning, socioemotional, physical and literacy–numeracy skills.

## Material and methods

### Study design and sample

The present study was performed as a secondary data analysis of the MICS 4 and 5, national data for Serbian Roma settlements, and public use data sets, conducted in Serbia in 2010 and 2014, carried out by the Statistical Office of the Republic of Serbia with support from UNICEF (available at http://mics.unicef.org/surveys). The surveys are designed to provide estimates of maternal and child health indicators at the national and regional level, and separately for Roma communities. MICS 4 and 5 capture both anthropometric and early child development data along with basic information on mothers, caregiving practices for young children and households. The surveys include a specific series of questions that capture several domains of child development and parental engagement. Roma mothers were asked to provide information on their children's age, sex, birth order, care and feeding practices, and parental, mother and father–child interaction.

Early child development measures were available for children aged 36–59 months. The sample included 1222 Roma children aged 36–59 months living with their biological parents, and the mother and her current partner/stepfather. Only 19% (235) of children were living with their biological fathers at the time of the survey. Out of 1222 cases (children), 13% had missing data, resulting in an uneven number of cases (*N*). Statistical analyses were conducted in R (4.0.2 and 3.11.0).

### Measures of paternal investment

*Direct paternal care*: the quantity of father's engagement in stimulating activities with his child was used as a proxy for paternal investment. Parental (mother and father) child engagement was reported by the Roma mothers, and describes the types and number of activities that a father (or mother) had engaged in with his child within the last three days. Six activities are considered central to early cognitive development and socio-emotional well-being (Sun et al., 2016): reading books or looking at picture books; telling stories to the child; counting or drawing with the child; singing songs/lullabies; taking the child outside the home, into a yard or park; and playing with the child. The total score of activities ranged from 0 to 6 points. The internal consistency of both the paternal and maternal investment in this sample was acceptable (*α* = 0.74). As no information was available on mothers’ partners’/stepfathers’ involvement with their stepchildren, in the analysis father figure presence was dichotomised as stepfather vs. biological father.

### Outcome variables: child growth and development

Height is a widely accepted indicator for health status, contingent upon genetic potential and the balance between nutritional and environmental stresses such as disease, especially in early life (Silventoinen, 2003). Short stature is frequently being associated with reduced reproductive success, and for women, greater offspring mortality, underweight and stunting (Nettle, 2002; Stulp et al., 2012). Still, in terms of reproductive success, being small might be disadvantageous in one environment but advantageous in another (Uggla & Mace, 2016; Gluckman et al., 2007).

Roma population-specific references that describe the physical development of children do not exist; where no local standard exists, the World Health Organization (WHO) has recommended the adoption of the international growth charts. Therefore, children's individual-level height-for-age *Z* (HAZ) scores and *Z*-scores below 2 standard deviations (–2SD, as an indicator of stunting) from the median of WHO's reference population were used as measures of physical growth. *Z-*scores are a commonly used indicator for children's nutritional status, as they measure the number of standard deviations from the median of the reference population by child age. Stunting (too small for age) captures the health of the child: if a child is stunted, it is very likely that he/she was exposed to malnutrition for a relatively long period (UNICEF, 2015).

Child development was assessed by the mother-reported Early Child Developmental scale, validated and used across a large number of low- and middle-income countries (Loizillon et al., 2017; UNICEF, 2019; McCoy et al., 2016; Miller et al., 2016), and modified by UNICEF for Roma settlements specifically (UNICEF, 2015). For Roma, an additional new measure has been developed, called the developmental score. The developmental score sums up the number of positive answers from the 10 questions on child development. Roma mothers answered a 10-item questionnaire on child development in four separate domains: literacy–numeracy, physical, social–emotional and learning. Responses indicating positive child development were added up, i.e. 0 means that no question was answered positively, while 10 implies that all of the questions were answered positively.

The four domains assess the following: (1) language/numeracy – identify letters, read simple words, identify numbers (the score ranges from 0 to 3); (2) physical – can pick up item with two fingers and does not often feel unwell (0–2); (3) socioemotional – gets along with other children, does not kick or bite other children (0–3); and (4) learning – can follow simple instructions, can perform simple tasks independently, is not easily distracted (0–3). The internal consistency of the Roma child development scale in this sample was fair with a Cronbach's alpha of *α* = 0.41, comparable with other recent studies using the same scale for early child development (Urke et al., 2018; Jeong et al., 2016).

### Covariates

To reduce the risk of confounding, additional variables were used in the analyses to account for maternal, paternal and child conditions. Maternal age at the time of the survey, parental literacy skills (can read the whole sentence/basic literacy or can read only part of the sentence/functionally illiterate) and household access to improved toilet facilities were used as proxies for socioeconomic status. Poor sanitation is closely related to low socioeconomic status, and it can be an important determinant of growth: germs from faeces cause diseases, especially diarrhoea, which can impair the overall nutrition of mothers during pregnancy and affect the early life of growing children (Spears, 2020). Other maternal variables included mother's investment and parity. Negative associations between parity and child quality have been reported across contemporary and historic populations, where maternal parity served as a rough measure for investment (Walker et al., 2008). Child variables included age in months and sex, to account for variation between younger and older children, boys’ and girls’ growth and development, and child's birth order.

### Statistical analyses

Descriptive statistics, Pearson product–moment correlation coefficient, chi-square and *t*-tests were used to describe and detect differences across variables based on the Roma family situation: living with biological father and mother, and living with biological mother and her partner/stepfather. Statistical significance was set at *p* =≤ 0.05.

Chi-square and *t*-tests were used to detect differences across all variables, based on Roma children's family situation: living with a biological father and mother, and living with a biological mother and her partner/stepfather (*n* = 1222). A chi-square test for independence was used for the following variables: height (dichotomous), sex, parental literacy, toilet type (improved vs unimproved) and development (dichotomous). A *t*-test was used for height and development in *Z-*scores, including four subscale segments, maternal and child age, and maternal investment. The Pearson correlation coefficient was used to assess the relationship between maternal and paternal investment.

Several multiple regressions were conducted to assess the association between father's investment and children's height (*n =* 184; in individual *Z-*scores) and father's investment and developmental score (*n =* 172), and four separate domains of child development (literacy–numeracy (*n =* 193), physical (*n =* 199), social–emotional (*n =* 183) and learning (*n =* 200)), in addition to whether the presence of a father vs. stepfather (father-like figure – 0, stepfather; 1, father) was associated with children's height (*n =* 933) and developmental score (*n =* 915).

Binomial logistic regression was performed to determine whether father's investment was associated with stunting of Roma children (HAZ −2SD; *n =* 182). Height was dichotomised as 0–<2 SD and 1–>2 SD, as a binary indicator for stunting.

In all regressions assessing the relationship between paternal investment and child outcomes, paternal investment **(**continuous) was an independent variable, while maternal age (continuous, in years), investment (continuous), parity (continuous), maternal and paternal basic literacy (0, illiterate; 1, litterate), household type of toilet facility (0, unimproved; 1, improved), child's age (continuous, in months), sex (0, girls; 1, boys) and birth order (continuous) were controlled variables. Father-like presence was independent variable, with all other variables as controlled. Unimproved sanitation/toilet facility included: flush/pour flush to somewhere else; pit latrine without slab/open pit; bucket; open defecation (no facility, bush or field); and improved (flush/pour flush to piped sewer system, septic tank, pit latrine and pit latrine with slab).

To assess predictors of paternal investment, a hierarchical multiple regression was used: in the first step, maternal investment and father's literacy were entered in the model. The second step involved the inclusion of the child characteristics: sex, age, height and birth order (*n =* 192).

## Results

The sociodemographic and anthropometry and development characteristics for the sample are presented in Table 1. The average age of the Roma children was 48 months (SD = 7.16). There was an excess of boys, and most children were third born. Only 19% of Roma children lived with their biological father. The average HAZ was −0.79 (SD = 1.66) while almost one-fifth of Roma children were stunted. The mean developmental score of Roma children was 6.64 (SD = 1.40), higher than that observed in recent studies assessing early child development using the same measures (Urke et al., 2018; Jeong et al., 2016). Out of the four domains of development, literacy/numeracy was the least developed among Roma children, as expected given the Roma low attendance rate of formal schooling.

**Table 1.** Sample characteristics

Characteristics	Mean (SD) or %	*N*
Child		
Sex (n = 1222)		
Female	48.4	591
Male	51.6	631
Age in months	48.17 (7.16)	1222
Height for age Z-score WHO	−0.79 (1.66)	1068
Height for age Z-score WHO (n = 1068)		
≤−2Z	19.1	204
>−2Z	80.9	864
Birth order	2.50 (1.66)	1222
Developmental score	6.64 (1.40)	1075
Literacy/numeracy	0.63 (0.86)	1167
Physical	1.64 (0.50)	1188
Learning	1.91(0.35)	1183
Social-emotional	2.42 (0.76)	1127
Live with biological father	19.2	235
Live with stepfather/mother's partner	80.7	987
Mother		
Age	27.10 (5.70)	1165
Parity	3.18 (1.59)	1165
Literacy (n = 1058)		
Literate	60.8	643
Illiterate	39.2	415
Maternal investment	3.25 (1.80)	1214
Father		
Age	30.20 (6.36)	
	30.20 (6.36)	224
Literacy (n = 235)		
Illiterate	6.8	16
Literate	93.2	219
Father investment	1.85 (1.70)	232
Household		
Type of toilet facility (n = 1218)		
Unimproved and open facility	16.2	197
Improved sanitation facility	83.8	1021

Both Roma mothers and fathers were relatively young; maternal parity was on average 3.18 (SD = 1.59; range 1–10). Almost two-fifths of mothers were illiterate, in contrast to 7% of Roma fathers. Investment was relatively low for both parents, but higher for mothers, at 3.25 (SD = 1.80), that for fathers, at 1.85 (SD = 1.70). In total, 69 or 29.7% fathers had no investment, or 0 activities with their children, followed by two activities (21.6%), one activity (16.8%), three activities (14.2%), four and five activities (9.9 and 3.4%, respectively), while only 4.3% (10) of fathers had high investment (or six activities). The majority of fathers played with their children (57.8%), took them to a park or yard (52.7%), sang (25.9%), told stories (22.1%) or engaged in name counting (15.3%), and only 10% read to their children. More than 14% of mothers living with their children's fathers (235) had no investment or 0 activities with their children; for all mothers, more than 10% (126 or 10.4%) had no investment, i.e. did not engage with their children at all (0 activities), while only 12% (150) engaged in all six activities.

The majority of households had access to improved sanitation/toilet facility; of those with unimproved facility (16.2%), 13.4% openly defecated (no facility, bush or field). The majority of mothers (87.4%) received social welfare (cash transfers/child allowances for the first four children in a family), and 90% of children did not attend kindergarten.

Statistically significant differences were found between maternal investment and parity. Maternal investment (*t*(1220) = 2.30; *p* = 0.02) was higher for Roma children living with stepfathers (*M* = 3.31, SD = 1.77) compared with children living with biological fathers (*M* = 3.01, SD = 1.84, effect size small, *ɛ*^2^ = 0.004).

Maternal parity was also higher (*t*(1220) = −4.20; *p* = 0.00) for children living with stepfathers (*M* = 3.66, SD = 2.00) than for children living with biological fathers (*M* = 3.07, SD = 1.50, effect size small, *ɛ*^2^ = 0.01). There was a moderate and positive correlation between maternal and paternal investment (*r* = 0.39, *n* = 232, *p* = 0.00): an increase in the mother's investment increases the father's investment and vice versa (a decrease in the mother's investment decreases the father's investment).

The relationship between paternal investment and Roma children's height for age *Z-*scores and stunting (HAZ < 2 SD) is shown in Table 2. On average, boys were 0.16 SDs shorter than girls (*β* = −0.16; 95% CI, −1.07, −0.06; *p* = 0.03). An increase in father investment was associated with an increase of child's height for 0.11 SDs (*β* = 0.11; 95% CI, 0.02, 0.114; *p* = 0.00). Roma boys were more likely to be stunted than girls (odds ratio, OR = 0.46; 95% CI = 0.21–1.01; *p* = 0.02), while children were less likely to be stunted if fathers’ investments were higher (OR = 0.40; 95% CI = 0.18–0.92; *p* = 0.03).

**Table 2.** Paternal investment and Roma children's height for age *Z-*scores and stunting (HAZ < 2 SD; *n =* 184 and *n =* 182)

	HAZ continious		HAZ −2	
	*β*	(95% CI)	OR	(95% CI)
*Child*				
Sex	−0.16*	(−1.07, −0.06)	0.46*	(0.21, 1.01)
Age (months)	0.14	(−0.002, 0.07)	1.00	(0.95, 1.05)
Birth order	−0.02	(−0.53, 040)	0.80	(0.23, 2.76)
*Mother*				
Age	0.08	(−0.05, 0.10)	0.98	(0.89, 1.08)
Basic literacy	0.02	(−0.50, 0.63)	0.98	(0.42, 2.31)
Maternal stimulation	0.07	(−0.09, 0.23)	0.99	(0.79, 1.25)
Parity	−0.14	(−0.32, 0.07)	1.13	(0.34, 3.79)
Type of toilet facility	−0.01	(−0.80, 0.67)	1.92	(0.74, 4.95)
*Father*				
Basic literacy	0.01	(−0.90, 1.07)	0.53	(0.10, 2.66)
Father investment	0.11*	(0.04, 0.16)	0.40*	(0.18, 0.92)

*p = ≤ 0.05.

OR, Odds ratio.

Predictors of paternal investment are shown in Table 3. There was a positive association between child's height and father investment, and maternal investment and father investment: an increase in child's height for 1 SD was associated with an increase in father investment for 0.16 SDs (*β* = 0.16; 95% CI, 0.01, 0.31; *p* = 0.04); and an increase in maternal investment of 1 SD was associated with an increase in father's investment of 0.44 SDs (*β* = 0.44; 95% CI, 0.09, 1.74; *p* = 0.03).

**Table 3.** Predictors of paternal investment (*n =* 192)

	*β*	(95% CI)
*Child*		
Height for age *Z*-score WHO	0.16*	(0.01, 0.31)
Age	−0.46	(−1.33, 0.11)
Gender	0.22	(−0.10, 1.58)
Birth order	0.32	(−0.07, 1.56)
*Mother*		
Maternal stimulation	0.44*	(0.09, 1.74)
*Father*		
Basic literacy	0.11	(−0.27, 1.72)

*p = ≤ 0.05.

WHO, World Health Organization.

Roma children's development and subscales/four segments (literacy/numeracy, physical, learning and socio-emotional) were not influenced by paternal investment or any of the variables examined (not shown). Associations between the presence of father vs. stepfather (father-like figure) and children's height and developmental score are shown in Table 4.

**Table 4.** Father vs. stepfather presence and Roma children height and development (*n =* 933 and *n =* 915)

	Height		Develpment	
	*β*	(95% CI)	*β*	(95% CI)
*Child*				
Sex	−0.01	(−0.22, 0.19)	−0.04	(−0.29, 0.06)
Age (months)	0.12*	(0.01, 0.04)	0.13*	(0.01, 0.04)
Birth order	−0.08	(−0.26, 0.11)	0.18	−0.003, 0.31)
*Mother*				
Age	0.11*	(0.01, 0.06)	0.14*	(0.01, 0.27)
Basic literacy	0.08*	(0.04, 0.49)	0.07*	(0.01, 0.39)
Maternal stimulation	0.11	(0.04, 0.16)	0.09*	(0.02, 0.12)
Parity	−0.32*	(−0.41, −0.13)	−0.04	(−0.21, 0.13)
Type of toilet facility	0.04	(−0.09, 0.49)	0.05	(−0.07, 0.42)
Father figure	0.03	(−0.15, 0.39)	0.03	(−0.13, 0.32)

*p = ≤ 0.05.

Children's height was associated with the child's age and maternal parity. An increase in child's age for 1 SD was associated with an increase in height for 0.12 SDs (*β* = 0.12; 95% CI, 0.01, 0.04; *p* = 0.00). An increase in maternal age for 1 SD was associated with an increase in height of 0.11 SDs (*β* = 0.11; 95% CI, 0.01, 0.06; *p* = 0.01). An increase in maternal parity for 1 SD was associated with a decrease in height of 0.32 SDs (*β* = −0.32; 95% CI, −0.41, −0.13; *p* = 0.00).

Roma child development was associated with the child's age, maternal age, literacy and investment. An increase in child's age of 1 SD was associated with an increase in developmental score of 0.13 SDs (*β* = 0.13; 95% CI, 0.01, 0.04; *p* = 0.00).

An increase in maternal age of 1 SD was associated with an increase in developmental score of 0.14 SDs (*β* = 0.14; 95% CI, 0.01, 0.05; *p* = 0.00). Maternal literacy increased a child developmental score by 0.07 (*β* = 0.07; 95% CI, 0.01, 0.39; *p* = 0.04). An increase in maternal investment of 1 SD was associated with an increase in developmental score of 0.09 SDs (*β* = 0.09; 95% CI, 0.02, 0.12; *p* = 0.01). The presence of a father or stepfather had no effect on either Roma children development or height.

To allow for comparisons with established standards for evaluating effect sizes, statistically significant results were put into SD units: the coefficient is the estimated SD change in *Y* associated with a one SD change in *X*1 (holding any other *X* variables such as *X*2, *X*3, etc. in the model constant). So multiplying the *β* coefficient by the SD of *Y* turns this into the change in *Y* in the original units associated with a 1 SD change in *X*1. This can be useful because the original units are usually much more meaningful. Thus, for father investment (see Table 2; *β* = 0.11, SD = 1.70), a 1 SD increase in father investment predicts 0.187 SDs of height, and a 2 SD increase in investment predicts 0.374 SDs of height. For children's height (Table 3; *β* = 0.16, SD = 1.66), a 1 SD increase in children's height predicts 0.27 SDs of father investment and a 2 SD increase in height predicts 0.54 SDs of father investment. For maternal stimulation (Table 3; *β* = 0.44, SD = 1.80), a 1 SD increase in maternal stimulation predicts 0.79 SDs of father investment, and a 2 SD increase in maternal stimulation predicts 1.58 SDs of father investment.

For child's age (Table 4; *β* = 0.12, SD = 7.16), a 1 SD increase in child's age predicts 0.86 SDs of height, and a 2 SD increase in child's age predicts 1.72 SDs of height. For child's age (Table 4; *β* = 0.13, SD = 7.16), a 1 SD increase in child's age predicts 0.93 SDs of development, and a 2 SD increase in child's age predicts 1.86 SDs of development. For maternal age (Table 4; *β* = 0.11, SD = 5.70), a 1 SD increase in maternal age predicts 0.63 SDs of height, and a 2 SD increase in maternal age predicts 1.26 SDs of height. For maternal age (Table 4; *β* = 0.14, SD = 5.70), a 1 SD increase in maternal age predicts 0.80 SDs of child development, and a 2 SD increase in maternal age predicts 1.60 SDs of child development. For parity (Table 4; *β* = −0.32, SD = 1.59), a 1 SD increase in parity predicts 0.51 SDs of height, and a 2 SD increase in parity predicts 1.02 SDs of child's height. For parity (Table 4; *β* = −0.04, SD = 1.59), a 1 SD increase in parity predicts 0.06 SDs of child development, and a 2 SD increase in parity predicts 0.12 SDs of child development.

## Discussion

This paper used nationally representative data from Serbian Roma communities to assess paternal investment (as defined in this paper) and its predictors, and the associations between paternal investment, stepfather presence and early child development and physical growth.

There were several main findings. In this Roma sample, parental investment in a number of direct care behaviours was relatively low, while maternal and paternal investments were positively correlated. Overall, almost 10% of children did not receive any investment from their parents, as measured in the present study. In line with other studies from low- and middle-income countries, almost one-third of Roma fathers did not interact with their children in the surveyed care behaviours, twice the proportion of unengaged Roma mothers: 29.7 vs. 14.2% (Jeong et al., 2016; Sun et al., 2016). There was a cross-sectional association between father's investment with child's height and stunting, while maternal investment and child's height appeared to be predictors of father's investment. There were no associations between father's investment and developmental score, and stepfather vs. father presence and Roma children outcomes. Instead, maternal and child characteristics explained both the overall development and height for Roma children.

Parenting practices may be influenced by a range of child and family characteristics, and political and economic development (Walker et al., 2011). In addition, local culture and traditions influence parenting behaviour: in many countries, childcare is culturally ‘mother centric’, with low participation from fathers (Hosegood & Madhavan, 2010). Thus, for instance, an average Serbian father spends only 11 minutes per day with his children, while only one in 20 fathers is fully involved in parenting (Republički zavod za Statistiku, 2020). The observed low paternal investment of Roma fathers may reflect the prevailing dominant patriarchal cultural pattern with a significant sex asymmetry in parenting (Čvorović & Coe, 2019). Additionally, bias in maternal reporting could account for this finding – studies have found that generally fathers tend to report significantly higher levels of involvement than mothers, contingent on numerous factors including ethnicity, the quality of the couple's relationship and the child characteristics (Charles et al., 2018).

Previous research found inconsistent relationships between father's direct care and children's height (Maselko et al., 2019; Jeong et al., 2016), but in this study, after adjusting for potential confounding factors, Roma fathers’ direct care was positively associated with their children's height. Fathers may contribute to their offspring's well-being in a number of ways: additive paternal care (‘cooperative care’ where both parents work together to care for children at the same time) can include complementing the mother's direct care or providing resources that allow for better nutrition (Gurven et al., 2009). Additive care may also include playing with a child or teaching (Starkweather et al., 2021). Paternal additive direct care may have similar impact to that of other allomothers: a child may receive a better overall gain and additive investments that thus could lead to better fitness outcomes (Emmott & Page, 2019). Given the positive correlation of Roma maternal and paternal investment, the overall investment may have positively affected children, resulting in better outcomes. However, the most obvious mode that paternal direct care can influence growth is via nutrition, i.e. feeding. MICS is cross-sectional in design, and measures of direct parental involvement did not include feeding practices, thus the results may be confounded by an unmeasured variable that correlates with paternal care. In this setting, another possible pathway that may reflect on the positive association between father investment and child height is reverse causality, such that fathers provided more care to taller/healthier children (Maselko et al., 2019). A Roma child's height, in addition to maternal investment, predicted father's investment: taller children had fathers who provided more direct care. In young children, height serves as a proxy for the cumulative effect of nutritional and health loads from conception (Frongillio et al., 2017). Thus, early childhood growth is an important measure of offspring quality, as it may influence future health and reproduction (Kramer et al., 2016).

Additionally, Roma child's height and the chances of stunting were influenced by the child's sex: Roma boys were more likely to be shorter and stunted than Roma girls, suggesting that they were more susceptible to nutritional inequalities than their girl counterparts of the same age. This pattern is consistent with previous findings where biased paternal investment was associated with the children's health/height but also sex (Alvergne et al., 2009; Čvorović, 2020a; Hagen et al., 2001). Among the Roma, parents may selectively invest in and support taller children (girls) who had the greatest potential to survive into adulthood and reproduce successfully, thus making the parents into grandparents, or in other words, enhancing the parents’ reproductive success (Čvorović, 2020a). Sex preferencing among the Roma in favouring girls is a common finding in Hungarian Roma groups as well (Bereczkei & Dunbar, 2002). Roma girls more often than boys engage in helping-at-the-nest, have a greater chance of marrying up the socio-economic scale and produce more surviving children compared with sons. In addition, having a high-quality (taller/healthier) daughter is regarded as a considerable advantage and a source of income among Serbian Roma who practice bride price (Čvorović, 2014).

Compared with other studies, where socioeconomic status and parental education were positively associated with early child development (Urke et al., 2018; Jeong et al., 2017; Paxson & Schady, 2010), in this setting, there was an apparent lack of relationship between child developmental score and also four domains of development (literacy–numeracy, physical, socioemotional and learning) with paternal investment and other variables.

In contrast to other studies, when a stepfather was introduced to the context, the presence of either father or stepfather had no influence on Roma children outcomes (Case & Paxson, 2001). Instead, child's age and maternal characteristics explained both the height and overall development for Roma children. Thus, older children were taller and had higher developmental score. Older children have higher reproductive value, and in poor populations, the later born children are often disadvantaged relative to earlier borns in nutritional status and growth, having higher morbidity and mortality (Lawson & Mace, 2009). In addition, maternal parity was negatively associated with child's height. Roma mothers with higher parity had children who were shorter than those of mothers with lower parity. Under poor conditions, numerous siblings may put children at higher risk of malnutrition, because of the discrepancy between family size and available resources. Maternal parity may also serve as a rough measure for investment: body size may be a proxy for a trade-off between offspring number and quality, or between the number and size of offspring, especially under resource-scarce conditions and in high-fertility settings such as with the Roma (Walker et al., 2008). Maternal parity was higher in stepfather households, but interestingly, unlike in other studies (Amato & Rivera, 1999; Lawson & Mace, 2009), maternal investment was higher for children living with stepfathers. One possible explanation as of why children living with stepfathers experience higher maternal investment may be that a stepfather is providing some extra resources, thus the mother may be experiencing a higher quality of life in this new relationship, both of which enable the mother to better provide for her children, including direct care. MICS does not include information on stepfathers’ resources, while maternal age, basic literacy skills and access to improved sanitation (as proxies for socioeconomic status) did not differ between mothers living with biological fathers vs. stepfathers. A more likely explanation is that Roma mothers in stepfather households may be compensating for the absence of a biological father by focusing more investment and attention on the children from former unions (Emmot & Mace, 2014). Higher investment and more attention could also serve as protection for any number of possible negative effects in the new home. This context could perhaps explain the lack of association of maternal and paternal characteristics and child development and maternal characteristics and child height: the poor conditions may have affected the ability of Roma parents to invest, and to make substantially enough investment to be detected or differentiate between children. Thus, mothers ‘get activated’ only in the presence of stepfathers (high risk), to protect their children and compensate for even the limited paternal investment. Roma children may be sensitive to this particular setting as well: child's age was associated with growth and development only in the stepfather's presence, thus younger and older sibling get to compete more in a stepfather's household, as there is an actual maternal investment to compete for.

Furthermore, maternal age was positively associated with both height and development: generally, older mothers tend to invest more in offspring, as they are less likely to have additional children and the investment focuses on the children they already have (Uggla & Mace, 2016). An additional explanation could be that this relationship reflects on maternal status within a family. Many Roma women face inflexible gender roles and expectations, and for many, having children in marriage is the only socially endorsed route for an improvement in status (Čvorović & Coe, 2019). A higher maternal status within a Roma family may include more power in decision-making concerning child's wellbeing such as diet and activities.

Additionally, there was a positive association of child's age, maternal investment, and literacy with children's overall developmental score. As a child ages, it is more likely to develop and learn skills and be ahead in development. The importance of maternal care behaviours and education for children's early development has been well described: parental support for learning (such as stimulating interactions and reading books) was found to be an important means through which parental education is associated with children's development (Sun et al., 2016; Jeong et al., 2017; Walker et al., 2011). In turn, maternal education can facilitate maternal investment and practices, as increasing levels of education lead to different thinking and decision-making patterns (Cutler & Lleras-Muney, 2010). This may be especially important to the Roma, given the high illiteracy rate among females: even low levels of education increase children's well-being and survival prospects (Sandiford et al., 1997; Čvorović, 2020a).

To the best of my knowledge, this is the first study to provide an account of paternal direct care as a proxy for investment, stepfather presence and child development and growth among the low-resource Roma. The study findings contribute new evidence of the drivers or lack of it of development and growth among children in marginalised ethnic populations, adding to the literature about paternal investment and child outcomes.

The majority of Roma children grow up in poverty, born to mothers with low education, and in homes with limited learning opportunities. In this context, parental investment was relatively low. Fathers have limited involvement in direct care of their young children and this involvement was not associated with child development. The presence of a father vs. a stepfather did not exert any influence on Roma children, insomuch as it did not have direct influence on the children's’ outcomes of growth and development. Roma paternal investment was low to begin with and father absence is likely to be less important in settings where fathers usually provide less support for their children (Lawson et al., 2017). In the presence of a stepfather, maternal and child's traits explained overall child development and growth. Maternal investment was higher for children living with stepfathers, thus mothers may be protecting their children from previous unions and compensating for paternal absence. Competition among Roma children – among younger and older siblings – was evident only when maternal investment was significant, in the presence of a stepfather. Thus, older children, born to literate, lower parity mothers of higher status and greater investment had better developmental and growth outcomes; girls were the preferred sex, as they were likely to be taller and less stunted than Roma boys, possibly owing to expected fitness benefits. Reverse causality emerged as the most likely pathway through which the cross-sectional, positive association of father direct care with child growth may manifest, such that Roma fathers tend to bias their investment towards taller, more endowed children, because of greater fitness pay-off.

The study had several limitations. The data were cross-sectional, limiting causal inferences between the variables under study. The developmental score and paternal investment were mother-reported and thus subject to biases: both measure how mothers perceived their child's development and their husbands’ involvement, and not actual child development and paternal behaviour. Furthermore, the reliability of Early Child Development scale was fair but similar to other recent studies, reflecting its limited adaptation to local culture and context (Urke et al., 2018; McCoy et al., 2017). The questions regarding literacy/numeracy have been shown to be too advanced for 3- and 4-year-old children (McCoy et al., 2016), this being particularly pertinent as regards Roma and other disadvantaged children where parental literacy is limited.

Additionally, to date, no specific growth references have been developed for the Roma, even though their Indian origin indicate an ethnicity impact to the anthropometric measures. Albeit the population-specific growth references may serve as a more biologically relevant measure of within-population assessment of children's growth (Kramer et al., 2016; Martin et al., 2019), the effects found in this study may be considerable, ranging from an approximately 0.33 SD in child's height to a more than 1 SD difference in paternal care (Winking & Koster 2015).

As child's growth and development are sensitive to available resources, and may be affected by aspects outside of direct family influence (Lawson et al., 2017; Winking & Koster, 2015), social assistance (cash transfers) may also have an effect on Roma family, including the growth and development of Roma children. For instance, in affluent settings, fathers tend to engage more in direct child care if their wives are employed and/or contribute a greater share of the couple's earnings (Raley et al., 2012). Roma mothers’ receipt of welfare could motivate Roma fathers to engage in direct child care: the majority of Roma women do not work (formal income leads to withdrawal of social benefits), but still support the family with cash transfers. Nevertheless, a recent study found that among Serbian Roma, receiving social assistance was associated with disintegration and a diminished role of the family (Čvorović & Vojinović, 2020), but whether welfare influence father–child relationships and child outcomes remains unexplored. Also, other potential confounders, such as parental height and health status, were not collected. To fully understand the effects of paternal investment on child outcomes, information should be collected directly from fathers and father-like figures and/or through observation, and include parental anthropometrics, as well as data on the presence of alloparents, which may have an effect on child outcomes, including growth (Sear & Mace, 2008).

## Ethical statement

The author asserts that all procedures contributing to this work comply with the ethical standards of the relevant national and institutional committees on human experimentation and with the Helsinki Declaration of 1975, as revised in 2008.

## Conflicts of interest

The author declares no conflicts of interest.

## Data Availability

Data are available from http://mics.unicef.org/surveys
